# Supplementary material for: Splice-Junction-Based Mapping of Alternative Isoforms in the Human Proteome
Source: Cell Rep. Author manuscript; Available in PMC 2020 Jan 15. (PMC6961840; doi:10.1016/j.celrep.2019.11.026)
Supplement: 2 [file NIHMS1546469-supplement-2.zip › DF1/PXD006675/LeftCoronaryArtery/LeftCoronaryArtery_17_MYH11_QCQGQAAQEAAGGGR.pdf]

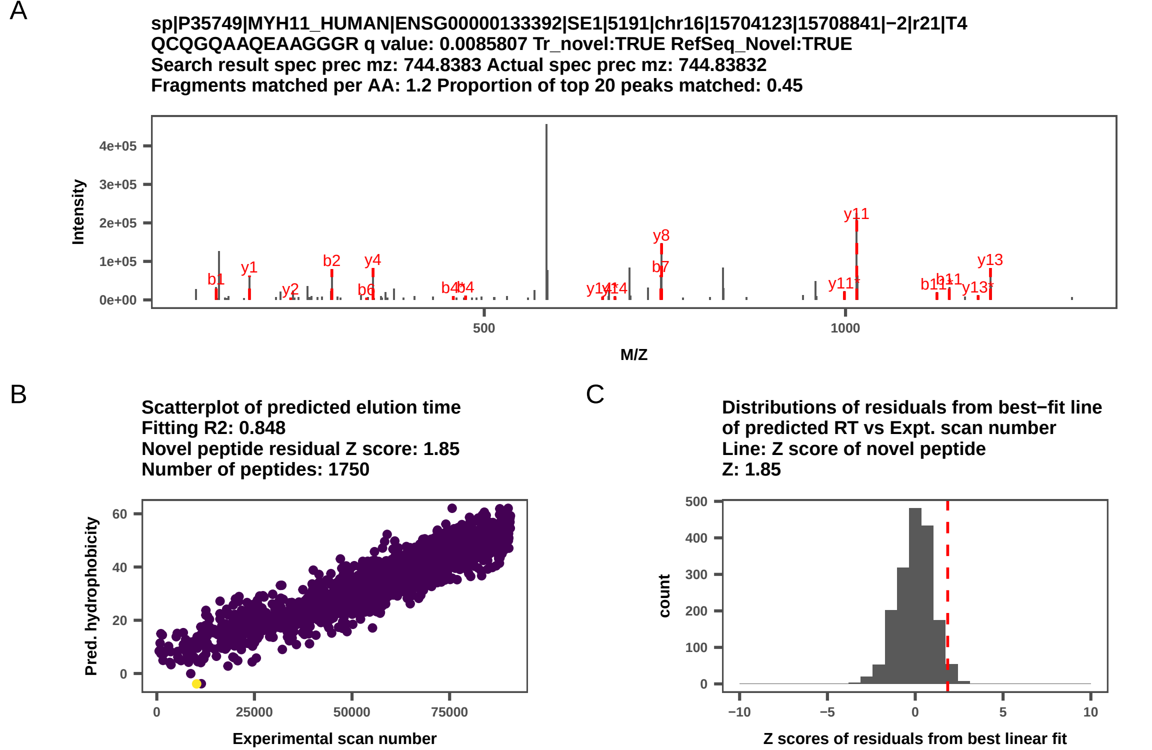
A
sp|P35749|MYH11_HUMAN|ENSG00000133392|SE1|5191|chr16|15704123|15708841|−2|r21|T4
QCQGQAAQEAAGGGR q value: 0.0085807 Tr_novel:TRUE RefSeq_Novel:TRUE
Search result spec prec mz: 744.8383 Actual spec prec mz: 744.83832
Fragments matched per AA: 1.2 Proportion of top 20 peaks matched: 0.45
Intensity
4e+05
3e+05
2e+05
1e+05
0e+00
b1
y1
y2
b2
y4
b6
y8
b7
y11
y11*
y13
y13*
b11
M/Z
500
1000
B
Scatterplot of predicted elution time
Fitting R2: 0.848
Novel peptide residual Z score: 1.85
Number of peptides: 1750
Pred. hydrophobicity
60
40
20
0
0
25000
50000
75000
Experimental scan number
C
Distributions of residuals from best−fit line
of predicted RT vs Expt. scan number
Line: Z score of novel peptide
Z: 1.85
count
500
400
300
200
100
0
−10
−5
0
5
10
Z scores of residuals from best linear fit
